# Elucidating the Landscape of Aberrant DNA Methylation in Hepatocellular Carcinoma

**DOI:** 10.1371/journal.pone.0055761

**Published:** 2013-02-20

**Authors:** Min-Ae Song, Maarit Tiirikainen, Sandi Kwee, Gordon Okimoto, Herbert Yu, Linda L. Wong

**Affiliations:** 1 Genomics Shared Resource, University of Hawaii Cancer Center, Honolulu, Hawaii, United States of America; 2 The Department of Molecular Biosciences and Bioengineering, University of Hawaii at Manoa, Honolulu, Hawaii, United States of America; 3 Cancer Biology Program, University of Hawaii Cancer Center, Honolulu, Hawaii, United States of America; 4 Cancer Epidemiology Program, University of Hawaii Cancer Center, Honolulu, Hawaii, United States of America; 5 Hamamatsu/Queen's PET Imaging Center, Queen's Medical Center, Honolulu, Hawaii, United States of America; Pontificia Universidad Catolica de Chile, Faculty of Medicine, Chile

## Abstract

**Background:**

Hepatocellular carcinoma (HCC) is one of the most common cancers and frequently presents with an advanced disease at diagnosis. There is only limited knowledge of genome-scale methylation changes in HCC.

**Methods and Findings:**

We performed genome-wide methylation profiling in a total of 47 samples including 27 HCC and 20 adjacent normal liver tissues using the Illumina HumanMethylation450 BeadChip. We focused on differential methylation patterns in the promoter CpG islands as well as in various less studied genomic regions such as those surrounding the CpG islands, i.e. shores and shelves. Of the 485,577 loci studied, significant differential methylation (DM) was observed between HCC and adjacent normal tissues at 62,692 loci or 13% (p<1.03e-07). Of them, 61,058 loci (97%) were hypomethylated and most of these loci were located in the intergenic regions (43%) or gene bodies (33%). Our analysis also identified 10,775 differentially methylated (DM) loci (17% out of 62,692 loci) located in or surrounding the gene promoters, 4% of which reside in known Differentially Methylated Regions (DMRs) including reprogramming specific DMRs and cancer specific DMRs, while the rest (10,315) involving 4,106 genes could be potential new HCC DMR loci. Interestingly, the promoter-related DM loci occurred twice as frequently in the shores than in the actual CpG islands. We further characterized 982 DM loci in the promoter CpG islands to evaluate their potential biological function and found that the methylation changes could have effect on the signaling networks of Cellular development, Gene expression and Cell death (p = 1.0e-38), with *BMP4*, *CDKN2A*, *GSTP1*, and *NFATC1* on the top of the gene list.

**Conclusion:**

Substantial changes of DNA methylation at a genome-wide level were observed in HCC. Understanding epigenetic changes in HCC will help to elucidate the pathogenesis and may eventually lead to identification of molecular markers for liver cancer diagnosis, treatment and prognosis.

## Introduction

Hepatocellular carcinoma (HCC) is one of the most common and lethal cancers in the world. The disease represents a significant health burden to our communities due to the lack of effective treatment [1], [2]. Chronic infection with hepatitis B virus (HBV) and hepatitis C virus (HCV), alcohol abuse, environmental and occupational toxins, as well as certain metabolic and immune disorders are risk factors of HCC [3], [4].

Sequential changes of normal hepatocytes to malignant cells are associated with altered gene expression reflecting accumulated genetic alterations, such as chromosomal instability (CIN) and DNA rearrangements, which have been extensively studied [5]. Nevertheless, the molecular pathogenesis of HCC remains poorly understood. Methylation of DNA cytosine residues at the carbon 5 position (5 mC) in cytosine-guanine dinucleotides (CpG sites) is a common epigenetic mechanism in eukaryotic DNA, playing an important role in regulation of gene activities. Aberrant DNA methylation is observed in many human cancers including HCC [6], [7], [8], in which global hypomethylation and specific promoter hypermethylation have been found as typical epigenetic changes involved in genomic instability and silencing of tumor suppressor genes, respectively [9], [10]. Furthermore, changes in cytosine methylation may also serve as molecular markers for prediction of tumor progression [11]. In HCC, aberrant hypermethylation of candidate genes such as *GSTP1*, *RASSF1A*, *APC* and *CDH-1* has been detected using methylation specific polymerase chain reaction (MSP), combined bisulfite restriction analysis (COBRA), and bisulfite sequencing techniques [12], [13], [14]. However, because the techniques for genome-wide analysis of methylation were introduced only recently, most studies have been of a limited scope with the analyses focusing on hypermethylation of tumor suppressor genes or on hypomethylation of oncogenes or methylation of repetitive elements such as LINE1 despite the fact that there are many more genes and DNA sequences in cancer that may develop aberrant methylation. Recently, genome-wide DNA methylation profiling in HCC was performed using the Illumina HumanMethylation27 BeadChip [14], [15], and these two studies identified many previously unknown regions and genes to be differentially methylated in HCC. However, further genome-wide analyses of DNA methylation with even higher resolution are still needed for better understanding of aberrant methylation in hepatocarcinogenesis.

In this study we used the latest version of the Illumina methylation microarray chip, the HumanMethylation450 BeadChip (Methylation450 BeadChip) to quantitatively analyze over 485,000 CpG sites in 27 HCC samples and 20 adjacent normal tissues. The analysis allowed us to confirm differential methylation in various known differentially methylated regions (DMRs) such as reprogramming specific DMRs (rDMRs), cancer specific DMRs (cDMRs) [7] and Enhancers (*in-silico* determined by Illumina), as well as to find potential new HCC DM loci. We also identified differential methylation in the genomic regions surrounding the promoter CpG islands, such as the shores and shelves.

## Materials and Methods

### Patient Specimens

The study was reviewed and approved by the Committee on Human Studies (Institutional Review Board) of the University of Hawaii at Manoa. All patients participating in the study gave written informed consent. Liver tissues were removed from the patients as part of a partial hepatectomy or liver transplant which was performed for hepatocellular cancer by a hepatobiliary/liver transplant surgeon (LW). No additional procedures were done other than what was clinically indicated for each patient. All operative procedures were performed at Hawaii Medical Center-East between 2009 and 2011. A total of 27 HCC tissues and 20 adjacent normal (non-malignant) tissues, including 19 matched pairs, were obtained. Histopathological evaluation was performed for each patient to confirm tumor content in the tissue. Relevant demographic and clinical information including patient age, HCC AJCC stage, sex, HBV and HCV infection status and alcohol use is shown in Table 1.

**Table 1:** Clinical and Pathological Characteristics of 27 HCC cases.

Clinicopathologic Characteristics	
Total case number	27
Median age (range)	66 (52–88)
Stage	
I	18 (67%)
II	3 (11%)
IV	1 (4%)
N/A	5 (18%)
Sex	
Male	20 (74%)
Female	7 (26%)
Viral status	
HBV-positive	9 (33%)
HCV-positive	10 (37%)
Non-HBV, Non-HCV	8 (30%)
Frequent alcohol use*	5 (19%)

* A person had more than 2 drinks a day for 10 years or more.

### DNA Extraction and Bisulfite Conversion

DNA was extracted from the tissue specimens using the AllPrep DNA/RNA Mini kit (Qiagen Inc, Valencia, CA). The quality and quantity of extracted DNA was examined by the NanoDrop-2000 (Thermo Scientific, Wilmington, DE). Bisulfite conversion of 500 ng of DNA was performed on each sample according to the manufacturer's recommendations for the Methylation450 BeadChip using the EZ DNA Methylation kit (Zymo Research, Irvine, CA). The treatment protocol included 16 cycles of denaturing at 95°C for 30 sec and incubation at 50°C for 60 min, as well as a final step of holding at 4°C.

### Methylation Analysis

HumanMethylation450 BeadChip (Illumina, San Diego, CA) was used to analyze the genome-wide DNA methylation profiles across 485,577 loci. These loci cover 96% of known CpG islands and 99% of NCBI Reference Sequence (RefSeq) genes, with an average of 17 CpG sites per gene distributed across the upstream of the transcription start sites (TSS)1500, TSS200, 5′ untranslated regions (UTR), first exon, gene body, and the 3′UTR. The 485,577 cytosine positions in the genome include 482,421 (99.35%) CpG dinucleotides, 3,091 (0.64%) CNG targets, and 65 (0.01%) SNP sites. Four µl of bisulfite-converted DNA was used for hybridization onto the Methylation450 BeadChip, following the Illumina Infinium HD Methylation protocol. This consisted of a whole genome amplification step followed by enzymatic end-point fragmentation, precipitation and resuspension. The resuspended samples were hybridized onto the BeadChips for 16 hours at 48°C. After hybridization, the unhybridized and non-specifically hybridized DNA was washed away, followed by single nucleotide extension using the hybridized bisulfite-treated DNA as a template. The Illumina iScan SQ scanner was used to create images of the single arrays and the intensities of the images were extracted using GenomeStudio (v.2011.1) Methylation module (v.1.9.0) software.

The data was normalized using the ‘Background Subtraction’ and ‘Normalization to Internal Controls’ methods offered by the Genome Studio software. First, the background subtraction value was derived from the signals of built-in negative control bead types for each channel, setting the background level at the 5% percentile of the negative controls in the given channel. Background was then subtracted from probe intensities in the same channel. If intensity becomes negative, it is set to 0. Secondly, the internal control probe pairs on the Methylation450 BeadChip were utilized for normalization. The normalization control probe pairs (over 90 of them) are designed to target the same region within housekeeping genes and have no underlying CpG sites in the probe. For normalization, probe intensity in the given sample was multiplied by a constant normalization factor (for all samples) and divided by the average of normalization controls in the probe's channel in the given sample.

The methylation score for each CpG site was represented by a Beta value calculated according to the normalized probe fluorescence intensity ratios between methylated and unmethylated signals. Beta values vary between 0 (fully unmethylated) and 1 (fully methylated). Every Beta value on the Methylation450 BeadChip is accompanied by a detection p-value indicating signals significantly greater than background and these detection p-values were used to remove noise from the data. Average Delta-beta values indicating the differential methylation between HCC and the adjacent normal tissue were calculated by subtracting the average Beta value of pooled HCC samples from that of pooled adjacent normal tissues.

A subset of differentially methylated (DM) loci (n = 8) found by the Methylation450 BeadChip analysis was selected for validation using Pyrosequencing. Bisulfite treated DNA was first amplified by PCR in the regions of interest using the PyroMark PCR kit (Qiagen) following the suggested protocol. Information on the predesigned and custom Pyrosequencing assays is in Table S5. One of the PCR primers was biotinylated to enable capture by Streptavidin Sepharose later in the protocol. The Pyrosequencing assay was performed on a PyroMark Q24 instrument (Qiagen) using the PyroMark Gold Q24 Reagents (Qiagen). Purification and subsequent processing of the biotinylated single-stranded DNA were performed according to the manufacturer's recommendations. The sequencing results were analyzed using the PyroMark Q24 software (Qiagen).

### Statistical Analysis of Differential Methylation

Partek Genomics Suite (Partek Inc., St. Louis, MO) was used for the statistical analysis of differential methylation. First, data quality control (QC) analyses on the normalized average Beta values generated by the GenomeStudio were performed. These included Principal component analysis (PCA) (Figure S1A), and graphing the sample histograms for signal distributions (data not shown). For the actual differential methylation analysis, a multivariate ANOVA, including factors such as tissue (tumor vs. normal), sex (male vs. female), virus infection (HBV or HCV positive vs. both negative), alcohol use and scan date, was performed to evaluate the contribution of these factors to differential methylation (Figure S1B). Since the 6-way ANOVA suggested tissue type to be the main significant contributor to differential methylation, we used 1-way ANOVA for tissue type by contrasting pooled tumor samples with pooled normals to determine significant differential methylation (DM) loci with unadjusted p-value cut-off as <1.03e-07 (corresponding to p-value <0.05 after Bonferroni adjustment). Heat maps were created using the Partek Genomics Suite. The Euclidian distance between the two groups of samples (tumors and normals) was calculated by the average linkage.

### Ingenuity Pathway Analysis (IPA)

The Ingenuity Pathway Analysis (IPA) program (http://www.ingenuity.com/index.html) was used to identifythe possibly affected gene networks, functional categories and canonical pathways related to HCC and liver function. IPA ranks gene networks by a score (-log (p-value)) that takes into account of the number of focus genes and the size of the network.

## Results

### Methylation Analysis

To explore the landscape of aberrant DNA methylation in HCC, we analyzed 27 HCC and 20 adjacent normal tissues (with 19 matched pairs) using the Methylation450 BeadChip. A total of five chips were used to profile 47 samples in three batches, and tumor-normal sample pairs were analyzed on the same chip to minimize experimental variation. After normalization of the data with internal controls and the background subtraction, Principal Component Analysis (PCA) was performed on the Beta values of the 485,577 loci to show methylation signal clustering of the samples by tissue type (shown as ellipses in Figure S1A). The results showed distinct overall methylation patterns between HCC and the adjacent normals. Moreover, the tumor group was less tightly clustered than the normal group, suggesting some heterogeneity in the methylation profiles among HCC. Next, we performed a 6-way ANOVA analysis with tissue type (HCC or adjacent normal), age, sex, alcohol, viral status and scan date as variables to assess the sources of variation in the methylation analysis. The most significant source of variation among these variables in the entire data of 485,577 loci was tissue type with other factors having a negligent effect (Figure S1B). Based on these results, 1-way ANOVA by tissue type was used for the final analysis.

### Global Methylation Profile in HCC

**Figure 1:**
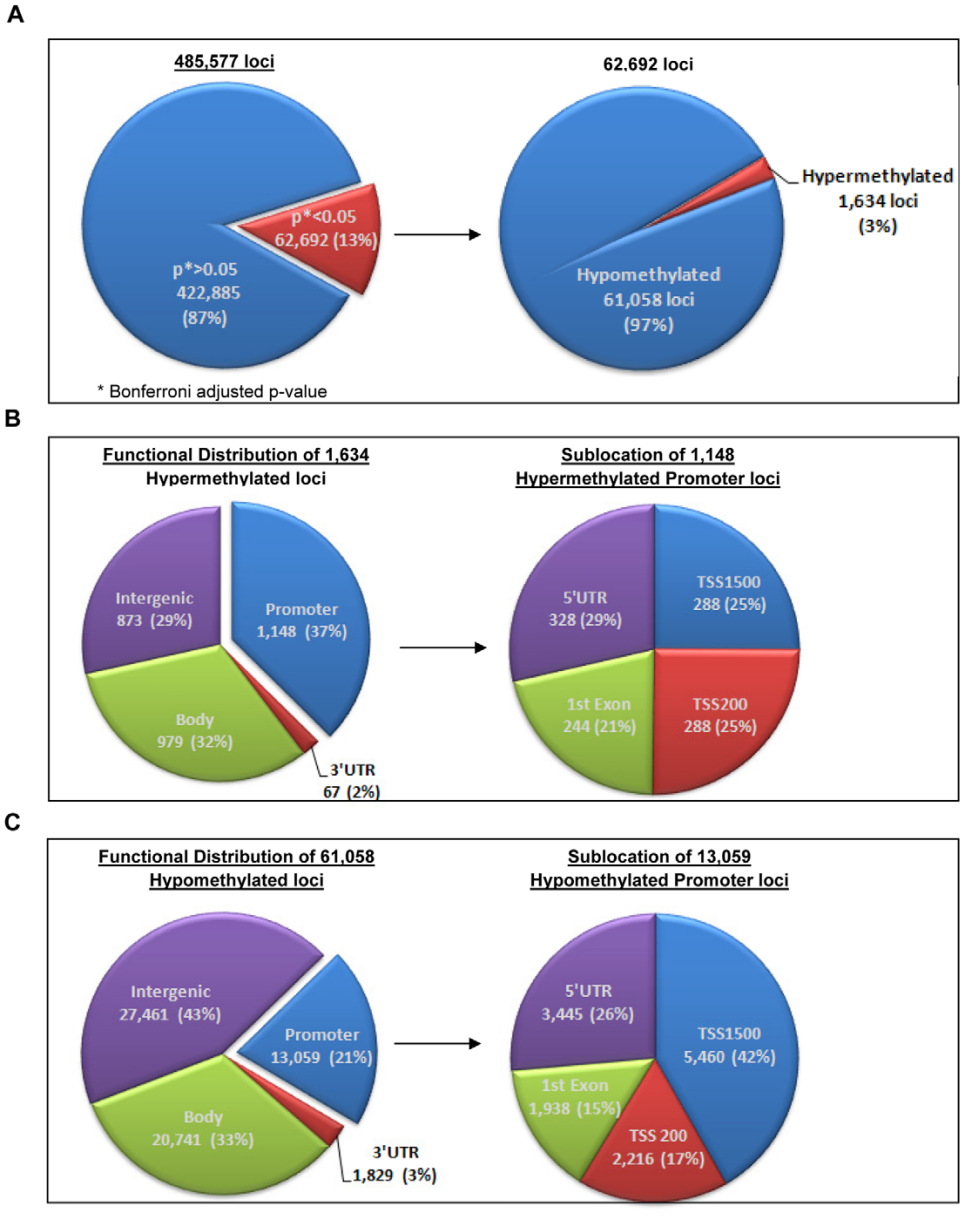
Genome-wide Methylation450 BeadChip DNA methylation portrait. **A. Left:** Significant differential methylation (DM) among the 485,577 CpG loci with a cut-off as Bonferroni adjusted p-value <0.05 (red) for statistical significance (blue; p-value >0.05). **A. Right:** The percentage of hypomethylated (hypo, blue) and hypermethylated (hyper, red) loci among the 62,692 DM CpG loci. **B.** Functional location of 1,634 hypermethylated loci and their sublocation in promoter regions including TSS1500, TSS200 or 1st Exon and 5′UTR. **C.** Functional location of the 61,058 hypomethylated loci and their sublocation in promoter regions.

Significant DM loci between HCC and adjacent normal tissues were observed in 13% of the CpG sites analyzed (62,692/485,577) (with unadjusted p<1.03e-07). Of these significant DM loci, 61,058 (97%) were hypomethylated (Figure 1A), compared to 1,634 (3%) loci that were hypermethylated. We further analyzed functional location of the DM loci in hyper- or hypomethylation groups with the notion that a CpG site can be in more than one functional location since a locus can reside in several transcript variants of the same gene or in different genes (therefore the total number of hyper- or hypomethylated loci and the sum of the loci in different functional locations don't match in Figure 1). Among the 1,634 hypermethylated DM loci, most (37%, a significant difference) were localized in the promoter regions which correspond to TSS1500, TSS200, 5′UTR and 1^st^ Exon, followed by 32% and 29% in the gene bodies and intergenic regions, respectively (Figure 1B). In contrast, hypomethylated DM loci (61,058) were mostly localized in the intergenic regions (43%) and gene bodies (33%) and were less likely to be in the promoter regions (21%) (Figure 1C). Another interesting observation of the hypermethylated loci is that they tend to locate in more than one functional location. The 1,634 hypermethylated genomic loci are located on 3000 possible functional loci, with an average of 1.84 functional loci for each genomic locus. The functional/genomic locus ratio for hypomethylated loci was only 1.03.

To understand how the observed DM distribution depends on the underlying normal patterns of DNA methylation, we characterized the patterns of DNA methylation in HCC and adjacent normal tissues separately (Figure S2). A heatmap showing methylation levels for 62,692 DM loci was generated by unsupervised hierarchical clustering (Figure S2A) and the methylation events for the 62,692 DM loci were further characterized by functional location (Promoter, Body, 3′UTR, or Intergenic) (Figure S2B). When looking at all DM loci (heat map) or by the functional DM loci, the normal tissue DM loci, with an exception of the promoter CpG islands, show consistently higher methylation levels in normals than tumors which contributes in apparent general hypomethylation in 97% of the DM loci in tumors.

### General Methylation Patterns in Promoter CpG islands and Surrounding Areas

**Figure 2:**
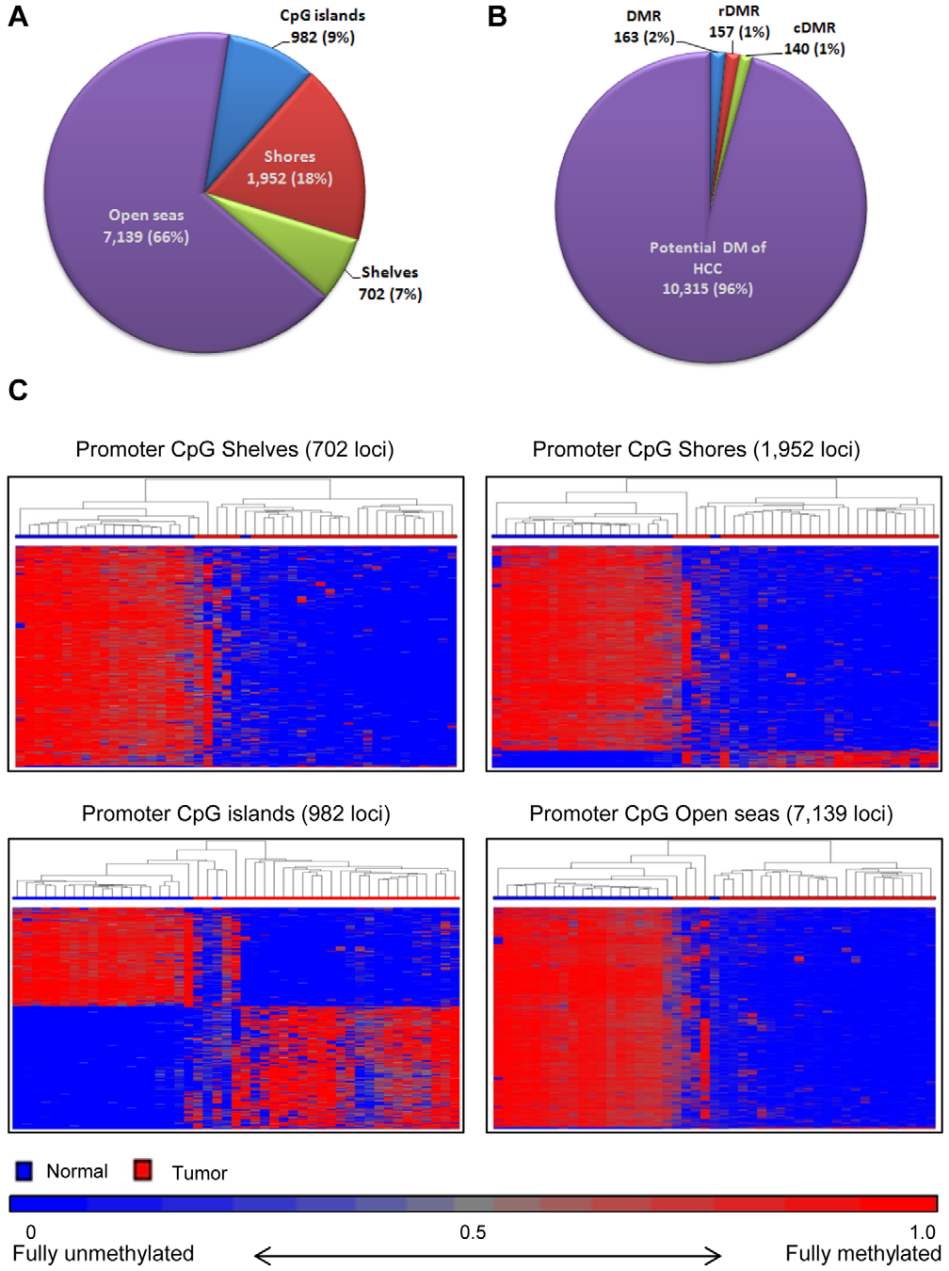
Methylation profiles of 10,775 promoter region DM loci by DMRs and in relation to CpG islands. **A.** Distribution of DM loci in CpG islands and the surrounding shore (0–2 kb from promoter CpG islands), shelf (2–4 kb from promoter CpG islands) and Open sea (other regions in promoter) DM areas. **B.** DM locus distribution by known differentially methylated regions (DMRs), reprogramming specific DMRs (rDMRs), cancer specific DMRs (cDMRs) and potential novel DMRs in HCC. **C.** Unsupervised hierarchical clustering of beta values for 702 Shelves, 1,952 Shores, 982 CpG island, and 7,139 Open seas loci (rows) in 47 samples (columns). Blue and red blocks on top of the maps represent 20 adjacent normal and 27 HCC tissues, respectively, while red for the loci represents hypermethylation and blue hypomethylation.

10,775 DM loci were located in CpG islands and surrounding areas. CpG islands in promoters as well as in the adjacent regions such as the shores (0–2 kb from the promoter CpG islands) and shelves (2–4 kb from the promoter CpG islands) have been found to be differentially methylated in cancer and the stem cells [7], [16], [17]. While most of the DM loci found in this study reside in the open seas, interestingly, differential methylation occurred in the shores twice as frequently than in the promoters (1,952 or 18% versus 982 or 9%) (Figure 2A). The actual methylation levels for each promoter CpG island and the surrounding regions are shown by heatmaps in Figure 2C. The branch lengths in the adjacent normal cluster were generally shorter than the branch lengths in the HCC cluster, indicating more heterogeneity in methylation levels among the tumor tissues, confirming the finding by PCA. The number of hypo- and hypermethylation events within the promoter CpG islands in HCC tissues were similar (45% and 55%, respectively), but significantly less hypermethylation was observed in the shores (8% of DM events), shelves (1%) and open seas (7%); hypomethylation being the dominant event in these regions.

### Methylation Patterns in Enhancers and Known and Novel DM Loci

Of the 10,775 promoter-related DM loci, 4% (460 loci) were in known DMRs, rDMRs and cDMRs that were established in colon cancer [7]. The remaining 10,315 DM loci located in 4,106 genes, on average 2.5 DM loci/gene, were newly identified and could potentially be HCC-specific (Figure 2B and Table S1). These HCC-related DM loci are harbored by genes which may have important biological functions in HCC. Our IPA of the 4,106 genes revealed significant enrichment for genes involved in ‘cell to cell signaling and cell death’ (p = 1.0 e-27). The top canonical pathways include ‘G-protein coupled receptor signaling genes’ (166 genes), ‘cAMP-mediated signaling’ (70 genes) and ‘cytokines in mediating communication between immune cells’ (25 genes). The top functions in hepatotoxicity include ‘liver hepatitis’ (31 genes), ‘liver cholestasis’ (11 genes), ‘liver damage’ (2 genes), ‘liver necrosis/cell death’ (4 genes) and ‘liver cirrhosis’ (33 genes) (Table S2).

**Figure 3:**
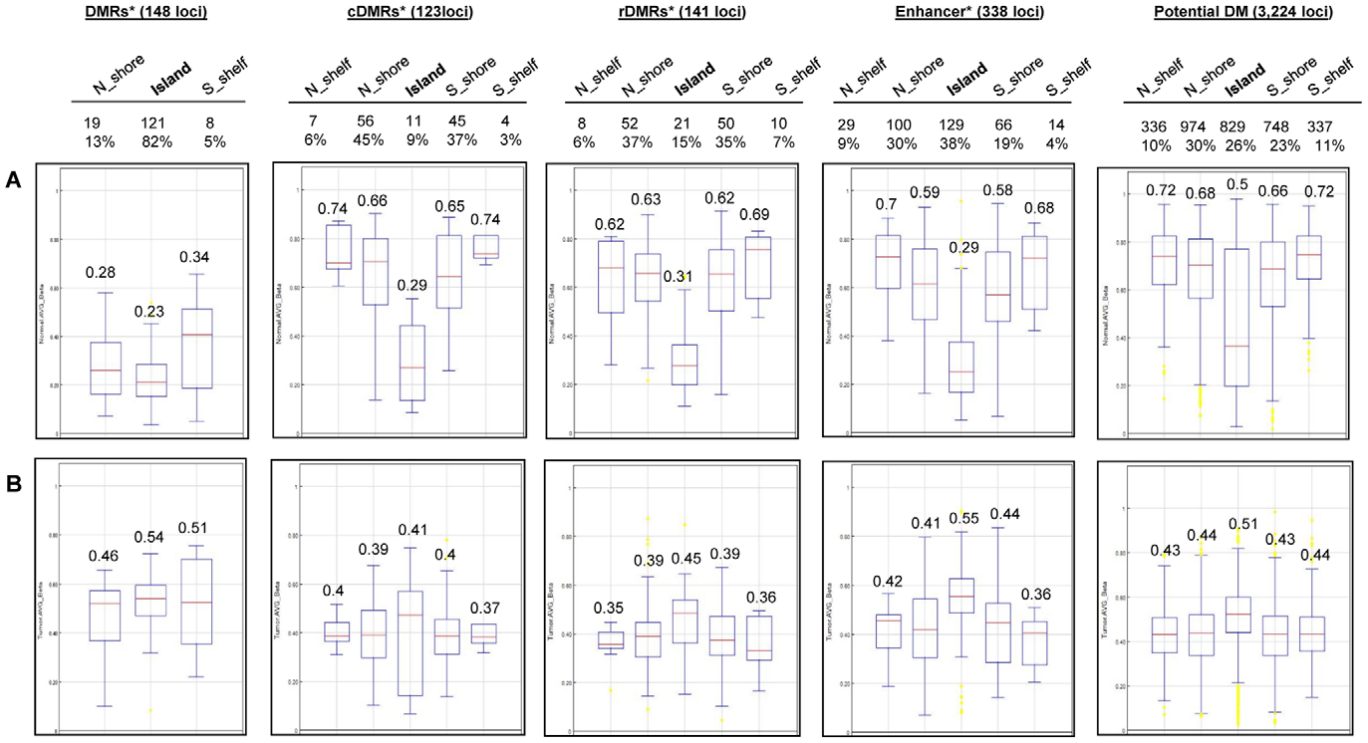
Distribution of promoter methylation levels. Genomic surroundings of the 10,775 promoter DM loci excluding the Open seas are shown **A.** in normals and **B.** in HCC tumors. The illustrative box plots present the median by a line in the box with the 25th percentile, 75th percentile and the range of the Beta values. Outlier values are shown with yellow color dots extending above or below the range markers. Density of functional loci on each genomic region is indicated on the top part of the figure. Averages of the Beta values are shown on each box plot. *previously known DMRs, cDMRs, rDMRs, Enhancers.

Because there is still limited understanding of the size and content of genomic regions involved in the promoter region epigenetic regulation in HCC, we focused next on the sublocation methylation levels of the 10,775 promoter-related DM loci including the known DMRs, cDMRs, rDMRs, and the potential new DMR loci as stratified by the promoter CpG islands, shores, shelves, and enhancers, but excluding loci in the open seas. The genomic regions were further classified into N_shelves (2–4 kb upstream of CpG islands), N_shores (0–2 kb upstream of CpG islands), CpG islands, S_shores (0–2 kb downstream of CpG islands) and S_ shelves (2–4 kb downstream of CpG islands). This analysis was done in the adjacent normal tissues and tumor tissues separately (Figure 3 and Table S3). Average Beta values of pooled samples for the DM loci within each region were used to examine the methylation levels. As seen in Figure 3, in the adjacent normal tissues, a higher density (number) of DM loci were observed in the shores than in the promoter CpG islands for all known cDMRs (9.2 fold), rDMRs (4.9 fold), the potential new HCC DMR loci (2 fold) and even for the DM loci within enhancers (1.3 fold), but not for the 148 general DMRs. The average methylation levels (Beta values) in the CpG islands of cDMRs, rDMRs and enhancers were approximately only half the levels of the loci in the shores (Figure 3A). In contrast, in HCC tissues, methylation levels across the CpG islands, shores and shelves were similar although the average methylation level seems to be slightly higher in the CpG islands than in the surrounding regions (Figure 3B).

### DM Loci in Promoter CpG islands

To further investigate the possible biological implications of differential methylation, we focused on a subset of 982 promoter DM loci (437 hypo- and 545 hypermethylated) that were located in the CpG islands within promoters. The average Beta and Delta-beta values between HCC and the adjacent normal tissues for the 982 loci are shown in Table S4. Among these loci, 80% (348/437) of the hypomethylated and 91% (494/545) of the hypermethylated loci had Delta-beta values ≥|0.2| which means greater than or equal to 20% methylation differences between tumor and normal tissues. The Methylation450 BeadChip can reliably detect a Delta-beta value of 0.2 with a lower than 1% false positive rate (http://www.illumina.com), giving confidence on findings with moderate Delta-beta values as well.

**Figure 4:**
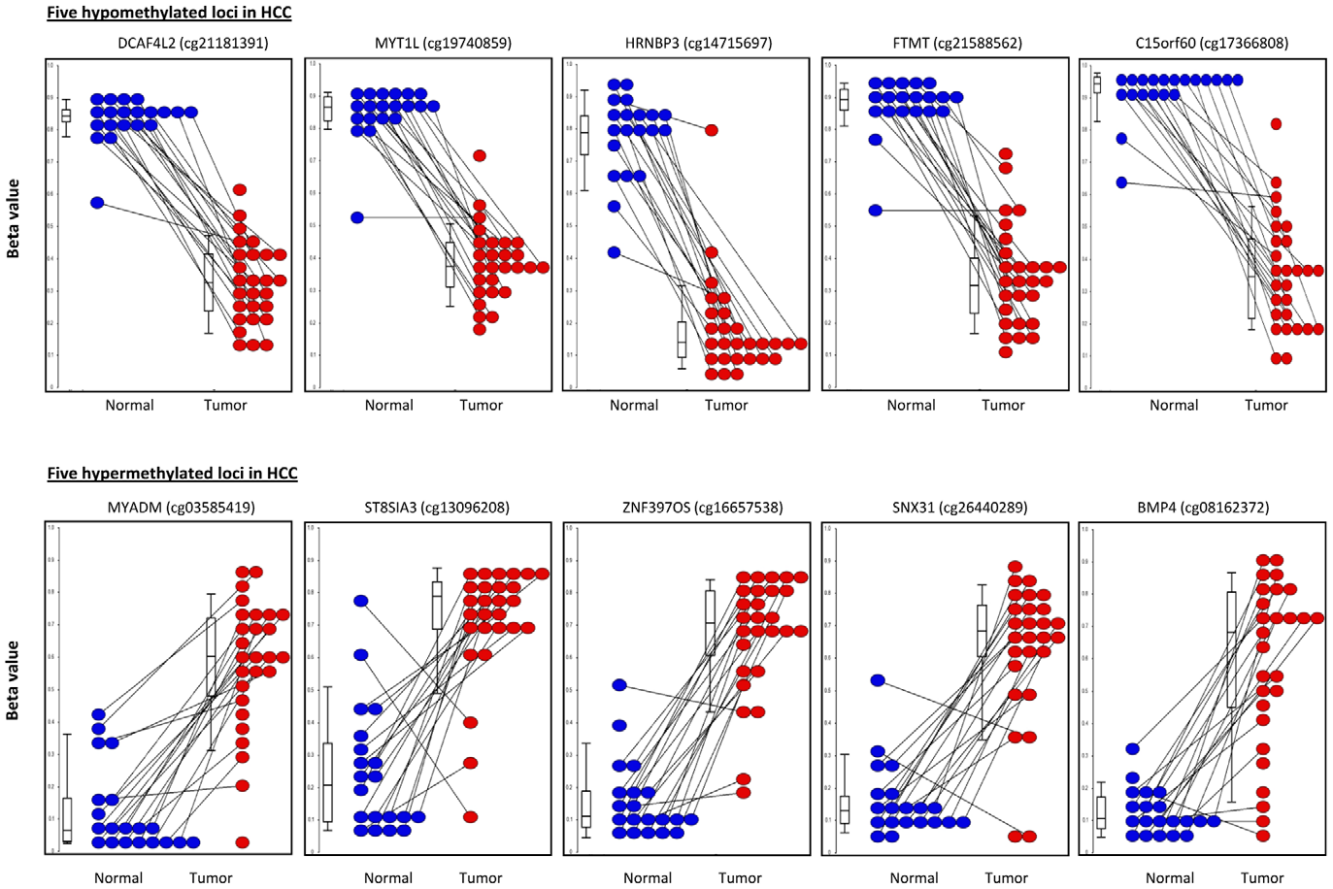
Dot plots of Beta values for 5 hypo- and hypermethylated loci among the 20 top based on the Delta-beta values from the tumor (n = 27, red) versus adjacent normal comparison (n = 20, blue). Each point represents the Beta value for an individual. The median Beta value for each locus and tissue type is indicated by a line inside each box-and-whisker within the graph. Paired samples are connected by a line.

The 437 hypo- and 545 hypermethylated promoter loci are co-located with 211 and 279 genes, respectively (Table S4). The DM loci ranked by the largest Delta-beta values are in Table 2, which indicates the gene corresponding to each locus, TargetID provided by Methylation450 BeadChip, Genomic location (GRCh37/hg19), Functional Location, Delta-beta values, and the Bonferroni adjusted p-values. Genes *C15orf60*, *DCAF4L2*, *GAB4*, *OR2B11*, *ACP1*, *SH3YL1*, and *C2CD4D* are covered by more than one locus. Moreover, TargetIDs cg06792448, cg10749741, cg25945732, cg03468349, and cg02339682 are localized in more than one gene. A high frequency of loci within TSSs (TSS1500 or TSS200, 12 out of 20 (60%)) was observed among the top 20 hypermethylated loci. In contrast, only 6 loci out of 20 (30%) were located within TSS200 among the top 20 hypomethylated loci. Interestingly, of the top 20 hypermethylated loci, three (genes *RIMS2*, *MYADM,* and *ZNF397OS*) were in known DMRs and four (*MTMR7*, *LPAR2*, *FOXE3*, and *SNX31*) were in the enhancer regions. *MYT1L* (adjusted p = 6.9e-14) and *FOXE3* (adjusted p = 9.0e-10) were found as the most significant hypo- and hyper-methylated genes between the HCC and adjacent normal tissues, respectively, while *NFATC1* (Delta-beta −0.6) and *RIMS2* (Delta-beta 0.55) showed the biggest methylation level differences between two groups among hypo- and hypermethylated genes, respectively. Overall, there was a high level of inter individual consistency in the differential methylation between the HCC and normal tissues. Beta values for 5 randomly selected hypomethylated and hypermethylated loci among the top 20 DM loci are shown by dot plots in Figure 4. The dot plots show consistent differential methylation of these loci between the HCC and the adjacent normal tissues for all patients except for a few outlier samples.

**Table 2:** Top 20 hypomethylated and hypermethylated loci in HCC based on Delta-beta values.

Hypomethylated top 20 loci in HCC						Hypermethylated top 20 loci in HCC					
Gene	TargetID	Genomic Location (GSCh37/hg19)	Functional Location	Delta-beta value	P-value^a^	Gene	TargetID	Genomic Location (GSCh37/hg19)	Functional Location	Delta-beta value	P-value^a^
**NFATC1**	cg02714192	Chr18:77201972	5′UTR	−0.60	4.8E-06	**RIMS2**	cg01566592	Chr8:104512858	TSS200	0.55	3.6E-08
**HRNBP3**	cg14715697	Chr17:77127487	5′UTR	−0.59	2.3E-12	**C2CD4D**	cg08703872	Chr1:151812435	5′UTR	0.54	2.5E-05
**C15orf60**	cg17366808	Chr15:73735476	5′UTR	−0.57	1.0E-11	**ACP1, SH3YL1**	cg20749741	Chr2:264178	TSS1500, TSS200	0.53	2.0E-05
**DCAF4L2**	cg02310286	Chr8:88886432	TSS200	−0.56	1.7E-10	**FOXE3^c^**	cg19809499	Chr1:47882265	1stExon	0.51	9.0E-10
**DCAF4L2**	cg11498870	Chr8:88886243	5′UTR, 1stExon	−0.54	4.2E-10	**FZD7**	cg23246885	Chr2:202899877	1stExon	0.51	3.4E-08
**FTMT**	cg21588562	Chr5:121187589	TSS200	−0.53	6.9E-12	**ZNF397OS^b^**	cg16657538	Chr18:32847566	5′UTR	0.51	1.6E-08
**C15orf60**	cg10480461	Chr15:73735509	TSS200	−0.52	3.7E-08	**ACP1, SH3YL1**	cg25945732	Chr2:264204	TSS1500, TSS200	0.50	1.0E-05
**MARCH1**	cg13078134	Chr4:165109478	5′UTR	−0.51	1.9E-08	**RNF135**	cg13204512	Chr17:29298184	1stExon	0.49	2.2E-08
**DCAF4L2**	cg21181391	Chr8:88886411	TSS200	−0.50	1.5E-14	**C2CD4D**	cg24425838	Chr1:151812421	5′UTR	0.49	3.2E-05
**ARHGAP8**	cg26523649	Chr22:45182193	5′UTR	−0.47	5.3E-05	**BEND6**	cg05022673	Chr6:56819429	TSS1500	0.48	1.7E-09
**MYT1L**	cg19740859	Chr2:2193641	5′UTR	−0.46	6.9E-14	**LPAR2^c^**	cg19306047	Chr19:19739407	TSS1500	0.47	1.7E-03
**DLGAP1**	cg26598649	Chr18:3880086	5′UTR, 1stExon	−0.46	1.1E-05	**MTMR7^c^**	cg12296772	Chr8:17271067	TSS200	0.47	1.9E-09
**GAB4**	cg02432860	Chr22:17489187	TSS200	−0.46	1.2E-05	**BMP8A**	cg10523966	Chr1:39957535	TSS1500	0.47	7.2E-04
**GAB4**	cg05945275	Chr22:17489130	TSS200	−0.45	5.1E-06	**SNX31^c^**	cg26440289	Chr8:101661921	TSS200	0.46	1.7E-05
**OR2B11**	cg05777962	Chr1:247614860	1stExon	−0.45	3.7E-11	**ST8SIA3**	cg13096208	Chr18:55019843	5′UTR, 1stExon	0.46	4.2E-05
**PMEPA1**	cg26681770	Chr20:56247302	5′UTR	−0.45	5.0E-03	**BMP4**	cg08162372	Chr14:54422925	TSS200	0.46	7.2E-04
**ZBTB46**	cg01548777	Chr20:62433998	5′UTR	−0.44	3.4E-05	**ZNF154**	cg21790626	Chr19:58220494	5′UTR, 1stExon	0.45	1.4E-04
**OR2B11**	cg14422922	Chr1:247614727	1stExon	−0.44	3.1E-10	**ACP1, SH3YL1**	cg03468349	Chr2:264164	TSS1500, TSS200	0.45	5.6E-05
**GML**	cg01972576	Chr8:143916273	5′UTR, 1stExon	−0.44	4.0E-10	**MYADM^b^**	cg03585419	Chr19:54369681	TSS1500, TSS200	0.45	2.4E-05
**RTL1, mir-136, mir-432**	cg06792448	Chr14:101349632	1stExon	−0.44	3.7E-03	**BEND6, DST**	cg02339682	Chr6:56819432	TSS1500, TSS200	0.45	1.1E-08

a Bonferroni Corrected p-value ^b^ DMR ^c^ Enhancer.

To explore the biological implications associated with the 982 promoter DM loci, we characterized the associated genes using IPA (Table 3). Twenty-six genes including *BMP4*, *CDKN2A* and *GSTP1* were involved in the top IPA networks related to‘Cellular development, Gene expression, and Cell death’ (p = 1.0e-38). Moreover, 15 genes were found to be involved in ‘Cellular movement, Cell cycle, Cellular compromise, and Cell-mediated immune response’ (p = 1.0e-17), and another 15 genes were identified in ‘Cellular development, Cellular function and maintenance’ (p = 1.0e-17), all of which were suggested as top networks in IPA. Cell cycle, proliferation, and cancer-related genes were also identified (Table 3).

**Table 3:** Biological functions of 10 IPA networks of the genes harboring 982 differentially methylated (DM) loci in the promoter CpG islands.

Molecules in Network		Score	Focus Molecules	Top Functions
**1**	Akt, *ARC*, **BMP4, CDKN2A**, *CTBP2*, **CYP24A1, DRD2**, E2f, ERK, **FOXD3**, *FTH1*, **GFI1, GNA11, GSTP1**, *H19*, Hdac, **HIST1H3A**, *HIST1H4A*, Histoneh3, **HMGA1, OXA11**, *ICAM1*, *IRS1*,**Jnk**, *MGAT3*, *MT1F*,*MYT1*, *NFATC1*, NFkB,**PAK1**, PI3K, RNA polymerase II, **RUNX3**, *TCL1A*, *TRPC3*	38	26	Cellular Development, Gene Expression, Cell Death
**2**	ABCB1, **ACP1, ADRB1**, AKAP5, CDH1, CLDN7, **COTL1**, CXCL2, **CYB5R2**, DKK1, DNMT3A,****DNMT3B, **DUOX1**, EED, **ELF4**, EPHA2, EZH2, FCGR2A, FOSL1, IL8, **IQSEC1**, JUN, *KRT81*,** LPAR2**, *MC5R*, *MYT1*, **NETO2**, Pkc(s), RAP1B, **SALL3**, **SDK1**, **SF3B14**, SMARCA4, SYK, Tgf beta	17	15	Cellular Movement, Cell Cycle, Cellular Compromise
**3**	**AKR1B1**, ANXA2, AR, AZGP1, CEBPB, **DST, FOXG1**, FOXO3,*FRG1*,GADD45B, HAS1, IL1B,****ITGB4, **KCNQ1**, KLF4, **LIG4**, **LRFN4, LRRK1**, *mir-1*, **NEDD4L, PFKP**, PGR, *PMEPA1*, PRKDC,****PTGS2, RPS19, SCNN1A, *SFMBT2*, **SLC22A16**, TAL1, TRAF6, TREM1,UGCG, XRCC4, XRCC6	17	15	Cell-mediated Immune Response, Cellular Development, Cellular Function and Maintenance
**4**	26s Proteasome, BIRC5, Caspase 3/7, **CHST2**, CXCL1, CXCL2, Dishevelled, **DSC3, FZD1, GNB4**, *GPR35*, Hsp90, *IFFO1*, Igm, IL13, Immunoglobulin, JAK1, *JAKMIP1*, *mir-154*, p70 S6k, *PDPN*,Pka, PML, RPS6, **SARM1**, *SCO2*, **SMPD3**, *SOCS5*, STAT6, TICAM1, TNF, TNFSF11, **TRPS1**, TYK2, VCL	15	14	Cell Death, Cellular Development, Hematopoiesis
**5**	AGTR1, **ATP2B4**, AURKA, Caspase, CBY1, CCND1, **CDKN2A**, CTNNB1, Cyclin A, DDX20, *E2F8*,**ENDOD1**, Estrogen Receptor, **FZD7**, GEMIN2, GEMIN4, Hdac, **HIST2H2BE, HMGA1, LDHB**, NCOA1, NRG1, *PABPC3*, PCDH11X/PCDH11Y, PPP3CA, **PRDM2, RASSF1**, SMN1/SMN2,SNRPD1, SNRPD2, **SNRPF**, SOX2, **TSC22D1**, TUBB2A, *YWHAE*	15	14	Cell Cycle, Cellular Assembly and Organization, RNA Post-Transcriptional Modification
**6**	ABCB1, **AFAP1**, APH1A, *APH1B*, **ASCL2, BMP4**, Calpain, CDK7, **CDKN2A**, CTSD, DDIT4,**** *DLGAP1*, **EBF1**, ESR1, ITGB1, *MAGEB1*, mir-21, mir-145, miR-21/miR-590-5p, MLL2, **MREG**, *NEK2*,NOTCH1, *PDE6B*, PML, PRDM5, PSEN2, Ras, RBBP5, SNAI2, **TMOD1**, TP53, TRIM28, TSG101, **ZNF274**	14	13	Cell Death, Cellular Development, Skeletal and Muscular System Development and Function
**7**	ACTA2, ANXA2, ARHGAP1, ATP2A2, **BMP4**, BMP6, BMPR1A, **CCNJ**, CREM, DHCR7, **DLX5**,****ERK1/2, FSH, ***GNAS***, GNB1, GPR56, hCG, IgG, ILK, *KRT18*, Lh, MED1, **NPR3**, P38 MAPK, PMAIP1,PTPase, PTPN1, **RAB31**, *RANBP1*, RAP1B, **RGS20, SRD5A2**, STAR, **TACSTD2**, *TRIM29*	12	12	Endocrine System Development and Function, Small Molecule Biochemistry, Lipid Metabolism
**8**	BCL2L11, CALML3, *CBFA2T3*, CCND2, CCND3, CDK2, CDKN1B, CHI3L1, **COL16A1**, DLL4,** EFNB2, FSCN1, FZD8**, *GLTSCR2*, IGFBP5, *IRS1*, KITLG, MMP3, **MYO10**, NOTCH1, PRKCA, *PSMA1*, PTEN, **PTPRN2**, SERPINE1, SMAD2, SNAI1, SNAI2, SPDEF, **SPDYA, SPINT2**, TGFB1, Vegf, VEGFA, VIM	12	12	Cellular Movement, Cellular Growth and Proliferation, Cancer
**9**	ABL1, *ANK1*, **BMP4**, CEBPD, DDIT4, **FKBP4**, GADD45B, GNAI2, *GPX1*, HNF4A, IGFBP1,****IRF4, **MIXL1, MTHFD2**, NANOG, NR3C1, **ONECUT1, OTX1**, POU5F1, PPARD, **PRKAR1B**, PTGES3, RARG, **RGS17**, RORA, RORC, **SCT**, Smad2/3, SOX2, SP1, TCF7, TRIB3, VIM, YY1, **ZNF148**	12	12	Cellular Development, Gene Expression, Tissue Development
**10**	ACO1, ADORA2A, **APBB2**, APP, *ARHGAP8/PRR5-ARHGAP8*, **CELSR1**, FUT1, IFNG, **IGFBP7**,****INSR, IRF7, JAK1, MAPK1, MAPK14, **MOV10L1**, P2RX7, **PAK1**, *PDE3B*, PIM1, PMAIP1, PML, RAC1, Ras, *RTN1*, **SECTM1**, SELE, SOD2, SPHK1, TCR, **TRIL, UCP2**, UGCG, VCL, VDR, ZAP70	12	12	Free Radical Scavenging, Cellular Movement, Cell Death

Italic and bold formatted genes represent hypo- and hypermethylated genes, respectively, with the bold italic formatted gene (*GNAS*) in network 7 having both hypo- and hypermethylated loci. The other genes are typically found in these networks in other studies, but not in our data set.

Interestingly, we found consistent opposite methylation patterns between two adjacent CpG islands in a known imprinted gene (*GNAS*, guanine nucleotide binding protein, alpha stimulating). There are two separate CpG islands in this gene with 5 hypomethylated loci located on CpG island 1 (at chr20:57,428,473–57,429,858) and 3 hypermethylated ones located on CpG island 2 (chr20:57,465,695–57,465,691) (Figure S3) in our HCC samples.

### Validation of DM loci by Pyrosequencing

**Figure 5:**
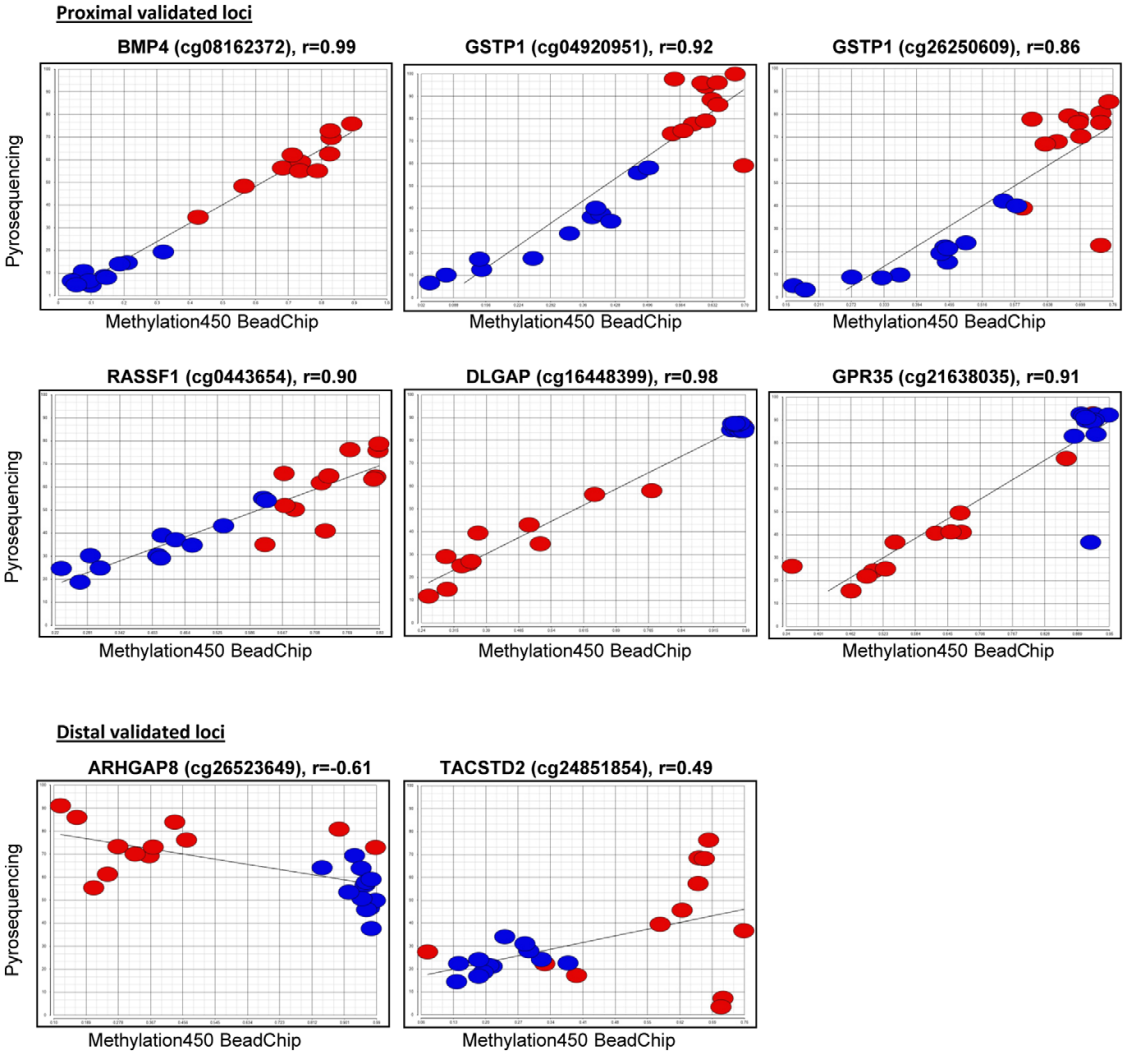
Validation results of 8 DM loci by Pyrosequencing. Gene name, Target ID (locus) by Illumina, and the correlation coefficients (r) are presented. Beta values for each individual HCC (red) and normal (blue) samples are presented by dots. The x-axis and y-axis indicate the Beta value from the Methylation450 BeadChip analysis and the methylation level by Pyrosequencing, respectively.

Eight DM loci identified by the Methylation450 BeadChip were selected for validation using pyrosequencing. Five hypermethylated loci harbored by four genes (*GSTP1*, *TACSTD2*, *BMP4* and *RASSF1*) and three hypomethylated loci in three genes (*ARHGAP8*, *GPR35* and *DLGAP1*) were validated using 7 assays with 12 tumor-normal tissue pairs from this study. Two DM loci were validated for *GSTP1* by pre-designed Pyrosequencing assays. Since there are limitations in designing assays for methylation loci due to high GC content, pre-designed assays were not always available for analyzing the exactly same locus that was assessed by the Methylation450 BeadChip. Nonetheless, since more than one locus with a significant p-value was observed in the genes, we selected the assays with the closest distance to the DM loci of interest to correlate the methylation results between the Pyrosequencing and the Methylation450 BeadChip. If the locus of interest was not covered by the assay, average methylation values of the analyzed CpG sites by Pyrosequencing were used for the correlation analysis. Five assays pre-designed by Qiagen and 2 previously used by other groups were used. Detailed information on the analyzed genomic region for each assay, the number of analyzed CpG sites by Pyrosequencing as well as the loci that show significantly differential methylation for a given gene on the BeadChip, but could not be technically validated, is shown in Table S5. For 5 of the genes, high correlations ranging from 0.86 to 0.99 were found between the Pyrosequencing and Methylation450 BeadChip results for both the exactly matched CpG sites (*GSTP1*, *RASSF1* and *BMP4*) and the closest CpG sites (*DLGAP1* and *GPR35*) (Figure 5, Proximal validatedloci). *BMP4* showed the highest concordance between the Pyrosequencing and the Methylation450 BeadChip (r = 0.99). We also observed that when the site of the Pyrosequencing assay was far away from the DM loci found by the Methylation450 BeadChip (about 0.9 kb for *TACSTD2*, 31 kb for *ARHGAP8*), the methylation status was not well correlated, indicating location specificity (Figure 5, Distal validated loci).

## Discussion

Aberrant DNA methylation has been shown to play an important role in tumorigenesis and cancer progression in many malignancies including HCC [18], [19]. A previous study focusing on DNA methylation profiles in HCC at a few selected loci of tumor suppressor genes and oncogenes found methylation changes in the promoters of 9 cancer-related genes, including *SOCS-1*, *GSTP*, *APC*, *E-cadherin*, *RAR-beta*, *p14*, *p15*, *p16 (CDKN2A)*, and *p73* [19]. New technologies of DNA methylation analysis utilizing microarrays now offer tremendous opportunities to study methylation at a genome scale. This approach can improve our understanding of the epigenetic mechanisms and the effect of DNA methylation on disease-associated molecular networks and pathways, beyond single genes.

In this study, we measured DNA methylation levels at 485,577 loci across 99% of RefSeq genes including 96% of the known CpG islands in 27 HCC tissues and 20 adjacent normals. After Bonferroni adjustment, we found statistically significantly differential methylation levels in 62,692 loci, 97% (61,058 loci) of which were hypomethylated, featuring general (or “global”) hypomethylation. Aberrant genome-wide hypomethylation is known to be linked to tumorigenesis, and our finding is in agreement with this notion. Previous studies also show lower levels of genome-wide 5′-methylcytosine contents in HCC tissues [20] and LINE-1 hypomethylation in the blood of HCC patients [21]. Furthermore, our finding of a disproportionate enrichment of hypomethylation events to the intergenic regions and gene bodies, rather than the promoters, bears resemblance to the findings of a previous genome-wide study on colon cancer [7].

Although several genome-wide methylation studies on HCC have been published recently [15], [22], [23], these studies have used the Illumina HumanMethylation27 BeadChip that includes much lower numbers of CpG loci than the Methylation450 BeadChip we used in this study. A recent study by Shen et al found methylation at 2,324 CpG loci (representing 548 hyper- and 1,290 hypomethylated genes) to be significantly different in 62 Taiwanese HCC cases (mostly HBV positive) compared to the adjacent normal tissues [15]. Among the 20 top ranked genes on their list, 14 of the hypermethylated genes (*DAB2IP*, *BMP4*, *CDKN2A*, *TSPYL5*, *CDKL2*, *ZNF154*, *ZNF540*, *CCDC37*, *NKX6-2*, *FOXD2*, *HIST1H3E*, *LYPD3*, *DNM3* and *RASSF1*) and 18 of the hypomethylated genes (*CCL20*, *AKT3*, *SCGB1D1*, *WFDC6*, *PAX4*, *GCET2*, *CD300E*, *CD1B*, *MNDA*, *CD1E*, *CYP11B1*, *KRTAP13-1*, *KLK9*, *KPNA-1*, *SPRR1B*, *SPRR1A*, *CCR6* and *OR51B4*) were also found with DM loci in our study. Furthermore, 14 out of the above hypermethylated genes were also identified by Ammerpohl et al. [22]. Since the Methylation450 BeadChip covers more CpG sites than the earlier version of BeadChip, we analyzed approximately sixteen times more loci than the previous studies, and each gene was covered by many more CpG sites (Table S6). Among our top ranked 20 hypo- and 20 hypermethylated loci within promoters (Table 2), two hypermethylated genes (*FTMT* and *GMT*) and six hypomethylated genes (*FOXE3*, *SH3YL*, *RNF135*, *BMP4, ZNF154* and *MYADM*) were also identified by Shen et al. [15]. Compared to the total of 1,490 DM loci found by the Illumina HumanMethylation27 BeadChip, we identified 62,692 DM loci using the HumanMethylation450 BeadChip. Locus wise, Our data is in 49% (735/1,490), 46% (685/1,490) and 57% (852/1,490) agreement with the previous studies by Ammerpohl et al. [22], Hernandez-Vargas et al. [23] and Shen et al. [15], respectively. The overlapping loci were 100% consistent in the direction of methylation changes (data not shown).

Previous studies reported statistically significant DM loci between HCC and normal tissues, but did not expand the analysis for a closer look at the structure of the DM genomic locations. However, relatively little is currently known about the significance of methylation changes between various genomic sublocations, i.e., promoter CpG islands, shores, shelves and enhancers. Irizarry et al. recently performed a genome-wide methylation study of colon cancer with a surprising finding that 13 times more altered methylation events were observed in the shores than in the promoter CpG islands [7].

In this study, we observed differential promoter methylation levels between the HCC and the adjacent normal tissues in the promoter regions including the TSS1500, TSS200, 5′UTR and 1^st^ Exon loci, methylation of which could directly regulate gene expression based on the current hypotheses. While the numbers of DM loci in the promoter CpG islands were quite similar for hypo- (45%) and hypermethylation (55%), significantly less hypermethylation events were found in the shores (8% of DM events) and shelves (1%) in the HCC compared to the adjacent normal tissues. This indicates that most of the promoter region hypermethylation events occur in the promoter CpG islands in HCC. Fittingly, looking at the level of methylation, normal tissues showed lower methylation levels in the promoter CpG islands than in the shores and shelves. In contrast, promoter CpG island methylation levels were higher than the levels in the shores and shelves in tumor tissues. Of the 10,315 promoter-related DM loci identified in this study, those that are not among the known DMRs, could potentially be novel DMR loci for HCC. The number of DM loci (mostly hypomethylated) was higher in the shores than in the promoter CpG islands for all the known rDMRs, cDMRs, as well as for the DM loci within enhancers and the potential new DMR loci, which bears resemblance to the finding of enrichment of colon cancer DM events on the shores rather than promoter CpG islands by Irizarry [7].

During development and carcinogenesis, methylation at each individual CpG site is established in a regional specific manner, with some regions that remain unmethylated while others become hypermethylated [24]. Similarly, we observed regionally consistent methylation patterns of the CpG loci within promoter CpG islands for the majority of the co-located genes (data not shown). Interestingly, we found one gene (*GNAS*) that had different methylation patterns in two separate promoter CpG islands. The hypomethylated CpG island is located upstream of a *GNAS* antisense RNA (*GNAS-AS1*) which suggests that the imprinting process may be regulated by these different patterns of methylation [25], [26].

For the evaluation of the possible biological implications of the aberrant methylation in the HCC tumors, we used IPA in order to identify pathways and cellular processes possibly disturbed by differential methylation of the affected genes. We found that the methylation changes observed in this study could be linked to the signaling networks of ‘Cellular development, Gene expression and Cell death’ (p = 1.0e-38). These networks include *BMP4* [27], *CDKN2A* [28] and *GSTP1* [29], genes that have been well studied in HCC before. Genes related to ‘Cell cycle, proliferation, and cancer’ were also indicated in our IPA analysis. Methylation at the CpG islands of promoters has been shown to directly correlate with gene expression [10], [30]. Our preliminary expression analysis of the 7 genes with promoter DM loci that we attempted to validate by pyrosequencing show at least partial correlation between promoter methylation and expression levels in 16 paired samples (data not shown). Of the 16 tumor-normal pairs with presumed hypermethylated promoter loci in HCC (in genes *BMP4, RASSF1,GSTP1*, and *TACSTD2*), 5, 6, 14 and 16 of the 16 HCCs had decreased expression of the gene, respectively. Of the pairs with presumed hypomethylated promoter loci (in *ARHGAP8, GPR35, and DLGAP1*), 10,11 and 12 of the 16 HCCs had increased expression of the gene, respectively. These results reflect the complexity of epigenetic regulation, since even that the differential methylation patterns were consistent between the tumors and normals for these particular genes (see Figure 5 for 5 of the genes); there was not always a direct correlation between the methylation and expression patterns.

Until recently, the importance of methylation changes beyond TSSs and CpG islands, e.g. in other gene regions such as gene bodies has been overlooked even though dynamic methylation patterns have been observed in these regions [31]. Since we have rich data from the Methylation450 BeadChip that allows simultaneous analysis of more than 480,000 loci on a genome-wide scale, we have detailed information on the methylation of CpG islands, TSSs, gene bodies and beyond in HCC. However, the possible heterogeneity of HCC tissue composition and the various risk factors of HCC may have substantial impacts on methylation. Therefore, it will important to evaluate methylation changes stratified by different risk factors of HCC, such as HBV and HCV infection. To gain a more comprehensive understanding of HCC methylation profiles, our future analysis will include specific risk factors of HCC, such as viral infection, alcohol drinking habits, disease stage, histology, and the presence of cirrhotic vs. non-cirrhotic surrounding tissue. We also aim to correlate the DM findings with gene expression beyond the current dogma, i.e. including DM loci that are not on the promoter CpG islands.

In summary, this is the first report to our knowledge to apply the Methylation450 BeadChip in HCC for assessing genome-wide methylation. Our current analysis focused on differential methylation patterns in various regions of the genome and especially in or near the gene promoters. Our main observations are: (1) General or “global” hypomethylation was observed in HCC, concentrating in the intergenic regions and gene bodies; (2) Discovery of 10,315 potentially new DMR loci for HCC with higher density in the promoter shores than in the CpG islands; (3) Higher frequency of hypermethylation events in the promoter CpG islands than in the shores and shelves; (4) Enrichment of promoter CpG island DM loci in gene networks of ‘Cellular development, Gene expression, Cell death, and Cancer’. Our findings from the genome-wide profiling of HCC may help to define the landscape of aberrant DNA methylation in HCC and give more depth to the observation of altered methylation in the promoter CpG islands and other related gene regulatory regions. Understanding epigenetic changes in HCC will help to elucidate the pathogenesis and eventually may lead to identification of molecular markers for disease diagnosis, treatment and prognosis.

## Supporting Information

Figure S1 **Principal Component Analysis (PCA)and sources of variation.** **A.** Two-dimensional PCA of DNA methylation data between HCC (red) and adjunct normal tissues (blue). x axis, first principal component (PC1); y axis, second principal component (PC2). **B.** Statistical significance of the different sources of variation in the methylation data estimated by a 6-way ANOVA model. F-ratio for each factor (source) represents the F-statistics for the factor/F-statistics for error (noise).
(TIF)

Figure S2 **Characterization of DNA methylation in adjacent normal and HCC tissues.** **A.** Beta values (ranges: 0–1) are shown for the 62,692 DM loci by unsupervised hierarchical clustering analysis. Redand blue blocks on top of the maps represent 20 adjacent normal and 27 HCC tissues, respectively. **B.** Beta values (y axis) on adjacent normal and HCC tissues are shown among the 62,692 DM loci by functional distribution. The box plots present the average methylation results, with median (indicated by a line in the box), the 25th percentile, 75th percentile and the range of the Beta values. Outlier values are shown with yellow color dots extending above or below the range markers.
(TIF)

Figure S3 **Genomic location of DM loci within** ***GNAS*** **promoter.** Genomic locations of five hypomethylated loci within CpG island 1 and three hypermethylated loci within CpG island 2 of the *GNAS* promoter are shown with average Beta-value graphs. The unadjusted and Bonferroni adjusted p-values are indicated for each locus on the table in the lower part of the figure.
(TIF)

Table S1 **Potential 10,315 DM loci in HCC.**
(XLS)

Table S2 **Top canonical pathways of the potential 10,315 DM loci in HCC.**
(XLS)

Table S3 **DMRs, rDMRs, cDMRs, Enhancers, and new DMRs of the 10,775 promoter DM loci** (**without the open sea loci**)**.**
(XLS)

Table S4 **List of 982 CpG island DM promoter loci in HCC.**
(XLS)

Table S5 **Information on the validated CpG loci and the pyrosequencing assays.**
(XLSX)

Table S6 **Shared CpG loci with Shen et al.**
(XLS)
