# Supplementary figures and images for: Elucidating the Landscape of Aberrant DNA Methylation in Hepatocellular Carcinoma
Source: PLoS One. 2013 Feb 20;8(2):e55761. doi: 10.1371/journal.pone.0055761 (PMC3577824; doi:10.1371/journal.pone.0055761)

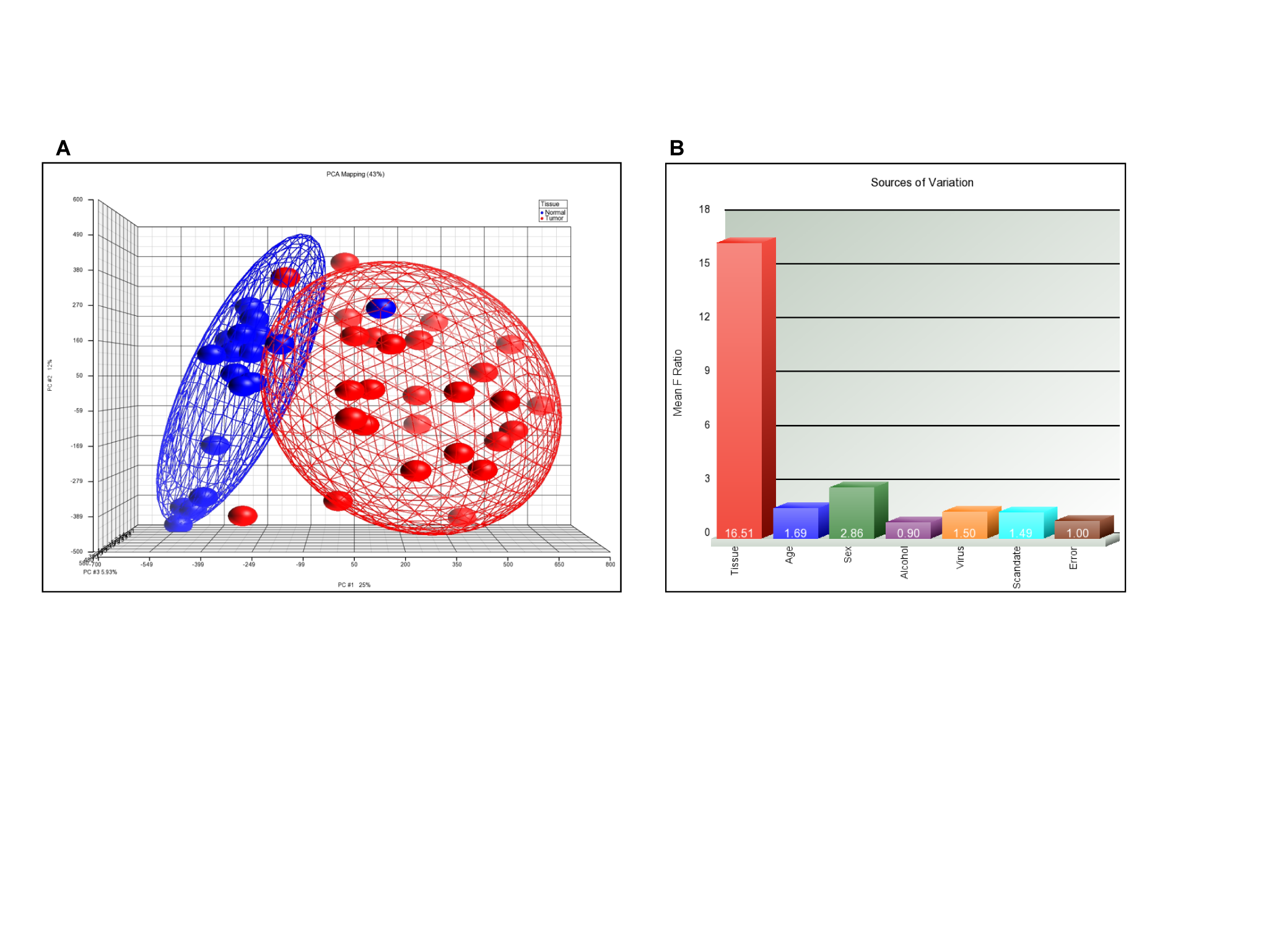

Supplement: Figure S1 — Principal Component Analysis (PCA)and sources of variation. A. Two-dimensional PCA of DNA methylation data between HCC (red) and adjunct normal tissues (blue). x axis, first principal component (PC1); y axis, second principal component (PC2). B. Statistical significance of the different sources of variation in the methylation data estimated by a 6-way ANOVA model. F-ratio for each factor (source) represents the F-statistics for the factor/F-statistics for error (noise). (TIF) [file pone.0055761.s001.tif]

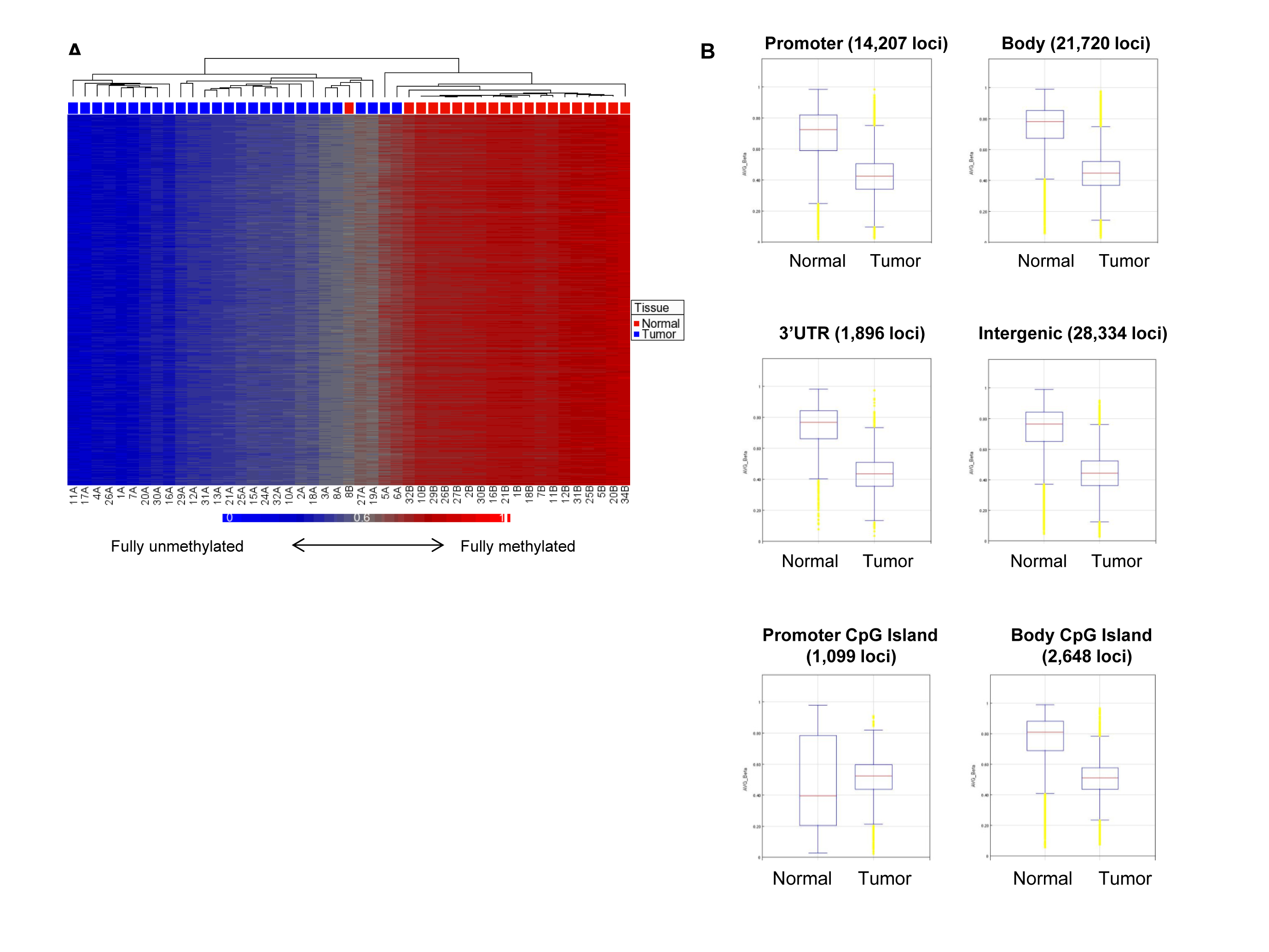

Supplement: Figure S2 — Characterization of DNA methylation in adjacent normal and HCC tissues. A. Beta values (ranges: 0–1) are shown for the 62,692 DM loci by unsupervised hierarchical clustering analysis. Redand blue blocks on top of the maps represent 20 adjacent normal and 27 HCC tissues, respectively. B. Beta values (y axis) on adjacent normal and HCC tissues are shown among the 62,692 DM loci by functional distribution. The box plots present the average methylation results, with median (indicated by a line in the box), the 25th percentile, 75th percentile and the range of the Beta values. Outlier values are shown with yellow color dots extending above or below the range markers. (TIF) [file pone.0055761.s002.tif]

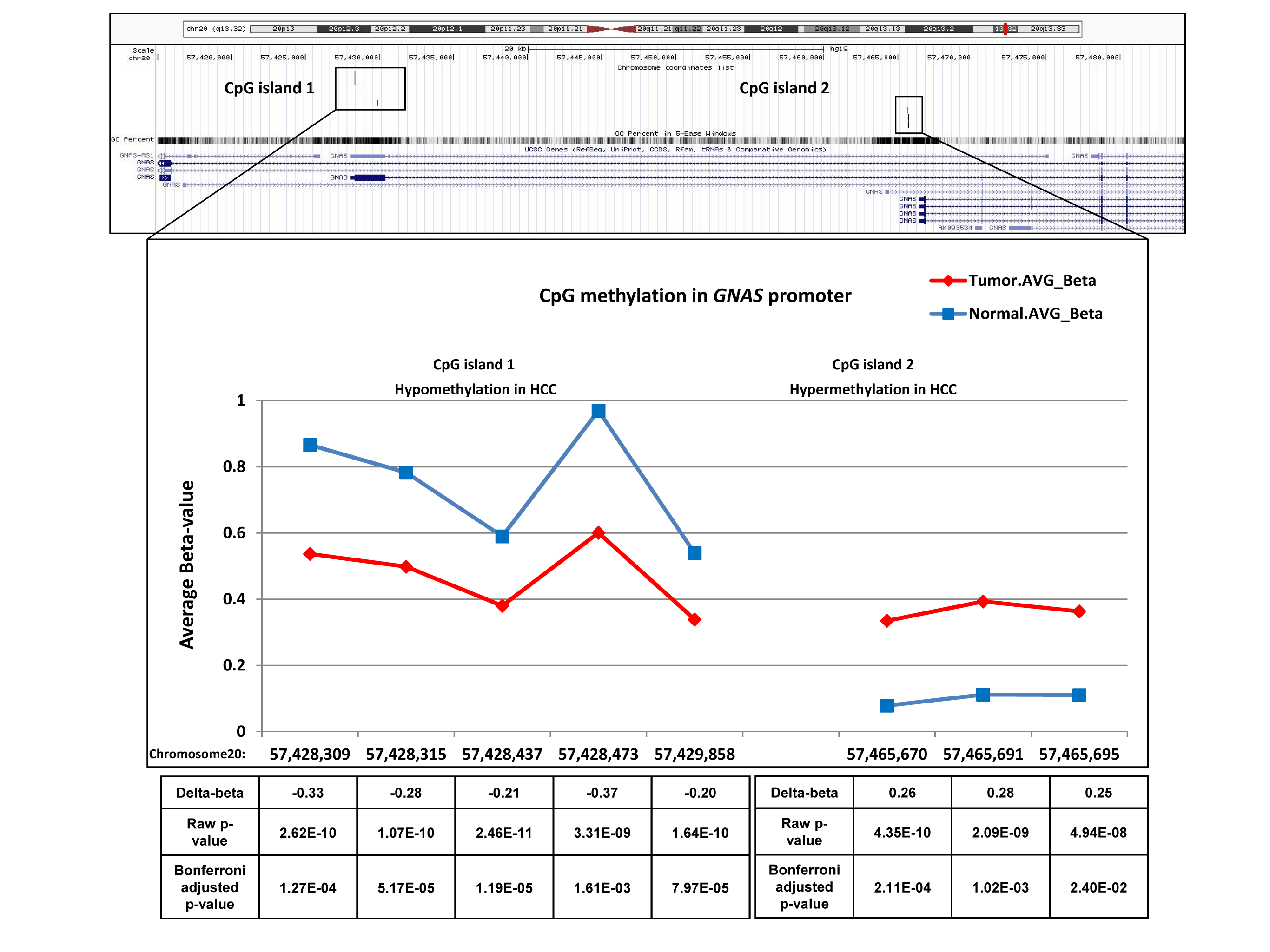

Supplement: Figure S3 — Genomic location of DM loci within GNAS promoter. Genomic locations of five hypomethylated loci within CpG island 1 and three hypermethylated loci within CpG island 2 of the GNAS promoter are shown with average Beta-value graphs. The unadjusted and Bonferroni adjusted p-values are indicated for each locus on the table in the lower part of the figure. (TIF) [file pone.0055761.s003.tif]
